# My Experience of Suprapubic Lithotomy

**Published:** 1901-04-27

**Authors:** Wheelton Hind

**Affiliations:** Senr. Assist. Surg. North Stafford Infirmary


### Hospital Clinics and Medical Progress.

### MY EXPERIENCE OF SUPRAPUBIC LITHOTOMY.

By Wheelton Hind, M.D., B.S., F.R.C.S., F.G.S., Senr. Assist. Surg. North Stafford Infirmary.

Very recently a most interesting discussion took place upon the suprapubic operation for stone, in the course of which some most remarkable statements were made, by operators of eminence, on the dangerous character of this operation. That such an opinion was held, and apparently with justice, according to the evidence adduced by those who condemned the operation, was a revelation to me, because the experience gained in my own practice and in the practice of my colleagues at the North Stafford Infirmary had taught me to consider this method of dealing with stone, at all ages, to be one of the safest and most satisfactory of operations. As far as I can gather by an analysis of the 43 cases operated upon in the North Stafford Infirmary during the last decade by my colleagues and myself, the mortality of this operation is exactly 0 per cent., and I find on inquiry that the same mortality obtains in our private practices.

In my own case I have for the last eight years always operated by the suprapubic method, and in patients whose ages vary from 73 to 4 years, on stones of all sizes, varying in number from 1 to 14, and I have operated on all cases of stone which came my way, so that the cases are not in any way selected. The average stay in bed is from 3 to 6 weeks, and I have seen no secondary trouble and experienced no difficulty during the convalescence. I cannot think that, judging from my facts, the operation is one that merits the name of a dangerous one, and I am at a loss to understand the heavy mortality and grave results quoted by others.

Suprapubic lithotomy to my mind is by far the simplest of all cutting operations of surgery, nor does it require extraordinary technical skill. The general surgeon, who is operating frequently, should find no difficulty whatever in the operation, the whole technique of which is most simple.

I have for some years now dispensed with the rectal bag, and simply, after evacuating the bladder, inject it with a lotion containing ac. boracis gr. x ad ℥i, sufficient in amount to cause bladder dulness to be felt a little way above the pubis. A rubber tube is tied round the penis to prevent evacuation, and a linear incision about 1½ inches long is made immediately above the pubis, the linea alba is found and divided, the fat round the bladder is torn through, a scalpel is then used to perforate the bladder (the edge of the blade presenting forwards) downwards obliquely behind the pubis, and a small incision, large enough to admit the tip of the left forefinger is made in the bladder wall. The finger then explores the bladder, defines the stone and serves as a guide for the smallest pair of forceps necessary for extraction. The bladder is then well washed out and a drainage tube inserted into the bladder, and the muscles and skin are united round the drainage tube. The tube is removed on the third day, entirely or gradually shortened, and I have never yet seen extravasation of urine round the wound.

In this operation the only point to be certain of is to avoid the peritoneum. The reflexion of this fold is easily made out and can be avoided, and if the stone be large the incision may be extended downwards as far as necessary without risk. Thus in technical dangers this operation has a great advantage over the lateral or median operation, when important organs, the rectum, the bulb and its vessels, the prostate, the orifices of the vasa deferentia may be wounded. The risk from hæmorrhage is very much less, no important arteries are cut, and incontinence of urine and impotence are not possible sequelæ. The risk of extravasation of urine into the surrounding tissues is much less when the wound is on the top of the bladder than when it is below. When the sphincter relaxes and the bladder contracts the tendency is greater for the urine to pass naturally than if there was a wound in the base of the bladder; for more force will be required to lift the water and to expel it from a higher opening than from a lower, and there is not a constant dribbling through the higher wound which obtains in the lower.

Where there has been cystitis the lavage of the bladder immediately is of great benefit, and this treatment can be repeated as long as is considered necessary by retaining the tube in the bladder, through the superpubic wound for that purpose. The open operation has the great advantage over crushing methods, that there is less irritation of the bladder, that there is good drainage, much less likelihood of a return of the complaint, because no splinters are made, all particles are removed, and no dilatation and consequent injury to the urethra, and the important ducts which open into it, takes place, and epididymitis is hardly likely to occur. But apart from this, and especially in inexperienced hands, the crushing operations are not at all without danger, *per se*; it is so easy to include a lax portion or fold of bladder wall, to miss an encysted stone, or to have miscalculated the size and hardness of the stone. Thus lithotrity is not an absolutely safe operation, and the death rate is certainly not so low as I find to be the case at the North Stafford Infirmary.

The interesting point to clear up is what are the causes of the very high death rate attributed to the operations in other hands. Do cases in other districts delay much longer before presenting themselves? But this fact would raise the mortality of all methods of treatment for stone—lithotrity among them. Strict asepsis is, I hope, universally adopted, and therefore should not be a varying factor in the case. Surely the personal equation of the surgeon cannot account for such different results, for as I have pointed out above, the operation is not extremely technical, and my results have been culled from the work of five surgeons, spread over several years, and therefore assisted by a frequently changing resident and nursing staff. Perhaps situation of hospital, hardness of race—our patients are colliers, ironworkers, potters and countrymen—may go for something, but whatever it is the fact remains that in my experience the suprapubic operation for stone in the bladder is one of the safest and most satisfactory of operations.

On looking over the last ten reports of the surgical work at the North Stafford Infirmary it is interesting to note the gradual way in which the suprapubic operation has superseded all others. During the first five years the lateral and median operations were more numerous than the suprapubic, but during the last five years the latter has almost entirely replaced all other methods. It is only fair, however, to state that the lateral and median operations have always been most successful at the North

Stafford Infirmary. I can find no record of a single death resulting from the operation, and I well remember a statement made to me on more than one occasion by the late Mr. J. Alcock, a pupil and surgeon to the institution for a very long period indeed, that he had never seen a death result from any operation for lithotomy. I think results such as these are well worth bringing before the profession.

